# First Report of *Polydrusus tibialis* Gyllenhal (Coleoptera: Curculionidae) Infesting Peaches in Northern Greece

**DOI:** 10.3390/insects16020192

**Published:** 2025-02-10

**Authors:** Stefanos S. Andreadis, Eleni I. Koutsogeorgiou, Emmanouel I. Navrozidis, Achilleas Kaltsidis, Dimitrios N. Avtzis

**Affiliations:** 1 Institute of Plant Breeding and Genetic Resources, Hellenic Agricultural Organization «Dimitra», 57001 Thermi, Greece; eikoutso@agro.auth.gr; 2 Laboratory of Applied Zoology and Parasitology, School of Agriculture, Aristotle University of Thessaloniki, 54124 Thessaloniki, Greece; 3 Department of Agriculture, School of Geosciences, International Hellenic University, 57400 Sindos, Greece; navrozidise@gmail.com; 4 Forest Research Institute, Hellenic Agricultural Organization «Dimitra», 57006 Vassilika, Greece; achikalts@gmail.com (A.K.); dimitrios.avtzis@elgo.gr (D.N.A.)

**Keywords:** peach, weevil, identification, biology, fruit deformation, leaf damage, univoltine

## Abstract

Peach [*Prunus persica* (L.) Batsch] is a major fruit crop particularly in Northern Greece, which is infested by several pests that threaten both yield and quality. In 2024, *Polydrusus tibialis* (Coleoptera: Curculionidae), a weevil species known to attack various fruit trees, was identified infesting peach orchards in the region of Pella, Northern Greece, for the first time. In this study, we present the initial report of *P. tibialis* as a pest in peach production, focusing on its morphology and biology. The monitoring was conducted in two orchards in the region of Pella using pyramid traps, and species identification was confirmed through morphological characteristics and DNA barcoding. The observations revealed that *P. tibialis* completes one generation per year, with adults emerging in early April and feeding on peach foliage and young fruit, causing economic damage. Larvae feed on root tissues, leading to wilting and stunted tree growth. The emergence of *P. tibialis* as a potential pest underscores the need for the immediate monitoring and development of integrated pest management strategies to protect peach cultivation in Northern Greece. Future research should focus on the pest’s ecology and effective control measures.

## Simple Summary

This study provides the first report of *Polydrusus tibialis* (Coleoptera: Curculionidae) infesting peach orchards in Northern Greece. It identifies the pest’s morphology and biology, with adults causing leaf and fruit damage and larvae feeding on roots, possibly leading to significant economic losses. This discovery highlights the need for monitoring and integrated pest management to protect peach cultivation in this region.

## 1. Introduction

Peach [*Prunus persica* (L.) Batsch] is a vital fruit crop in Greece, particularly in the northern regions where the climate and soil conditions are conducive for high-quality production [1]. In the southern region, peach cultivation is limited, mainly due to the lack of chilling conditions [2,3]. Greece is among the leading producers of peaches in Europe, with extensive cultivation primarily in the regions of Central Macedonia, Western Macedonia and Thessaly [4]. However, peach cultivation is constantly threatened by various insect pests, which can cause significant economic losses. Common insect pests of peach in Greece include the Mediterranean fruit fly (*Ceratitis capitata* Wiedemann), the peach twig borer (*Anarsia lineatella* Zeller), the oriental fruit moth [(*Grapholitha molesta*) (Busck)], the summer fruit tortrix moth [*Adoxophyes orana* (Fischer von Rösslerstamm)] and the green peach aphid (*Myzus persicae* Sulzer) [4,5,6,7,8].

During the previous growing season, a new potential pest, *Polydrusus tibialis* (Gyllenhal), has been observed in peach orchards in Northern Greece. *Polydrusus tibialis* is a weevil species (Curculionidae) known for infesting various fruit trees, but its presence in peach orchards has not been previously documented in this region. It was found infesting peaches in Croatia [9], and its presence has also been recorded in other host plants, such as Cretan maple (*Acer sempervirens*), pears (*Pyrus communis*), hawthorn (*Crataegus* sp.) and oak (*Quercus* sp.) in the regions of Attica, Central Greece, Central Macedonia, Crete, East Macedonia and Thrace, Epirus, Ionian Islands, Peloponnese, West Greece, and West Macedonia [10], as well as cherries and plums in the Prefecture of Pella (personal observations).

This report aims to present the first evidence of *P. tibialis* infesting peach crops in Northern Greece and to provide a comprehensive overview of its morphology, taxonomy, and biology, since the introduction of *P. tibialis* as a potential pest of peach would add to the existing burden of pest management in these orchards.

## 2. Materials and Methods

### 2.1. Locations

This study was conducted from early April up to late July 2024 in two peach orchards (Aravissos and Edessa) of the regional unit of Pella (Table 1), a region in Northern Greece with a high fruit-orchard production. Both orchards were managed with similar IPM practices, complying with EU standards of pesticide residues below the respective MRLs [11].

### 2.2. Monitoring Traps

To estimate the relative density of *P. tibialis*, unbaited black-standing monitoring traps (≈120 cm tall and ≈50 cm wide at the base) made of heavy-duty corrugated plastic [Tedders (Pyramid) Trap, 6/CS, Great Lakes IPM^TM^, Vestaburg, MI, USA] were placed in both locations. More specifically, two monitoring traps, at least 10 m apart, were deployed in each location at the perimeter of the orchards, as per our previous year observations of a leaf infestation in the aforementioned areas. The traps were inspected weekly from early April to late July.

### 2.3. DNA Barcoding

Adults captured in pyramid traps were immediately placed in 2 mL tubes with alcohol (>70%) and then shipped to the Laboratory of Forest Entomology (Forest Research Institute—Thessaloniki, Greece). Due to the close morphological resemblance with other *Polydrusus* species, DNA extraction was performed using the front leg of a specimen with PureLine^®^ Genomic DNA kit (Invitrogen, Waltham, MA, USA). Polymerase Chain Reaction (PCR) amplification was run in 25 μL with DreamTaq DNA Polymerase (Thermo Fisher Scientific, Waltham, MA, USA) and using LCO-HCO primers [12] that amplify a 658 bp long locus of the mitochondrial cytochrome oxidase subunit I (COI) gene. The PCR procedure included an initial denaturation at 94 °C (3 min), followed by 45 cycles of 30 s at 94 °C (denaturation), 30 s at 47 °C (annealing), and 1.5 min at 72 °C (extension), whereas the final extension period at 72 °C was run for 5 min [13]. The PCR products were cleaned up enzymatically using the ExoSAP-IT TM PCR Product Cleanup Reagent (Thermo Fisher Scientific, Waltham, MA, USA) and then shipped to Cemia Company (Larissa, Greece) where they were sequenced in an ABI3730XL automated sequencer using both the forward and reverse primers of the PCR in order to avoid erroneous base calling. The sequences were initially visualized with the Chromas Lite version 2.6.6 software and then aligned with ClustalX version 2 [14]. Finally, species identity was defined based on the results of a nucleotide BLAST query in the NCBI GenBank using the obtained sequences.

## 3. Results and Discussion

The identity of the weevil was initially concluded by the distinct morphological trait (spurs on the tibia of the legs) and subsequently verified by the 100% sequence similarity with the only available DNA barcode of the species (KC784117) [15], confirming that *P. tibialis* is infesting peach orchards in the regional unit of Pella (Northern Greece). The genus *Polydrusus* is part of the subfamily Entiminae that falls within the Curculionidae family [16]. *Polydrusus tibialis* was first described by Gyllenhal in 1834 and has since been recorded in various parts of Europe, feeding on a variety of different hosts that include deciduous trees and shrubs [17].

### 3.1. Morphology

**Figure 1:**
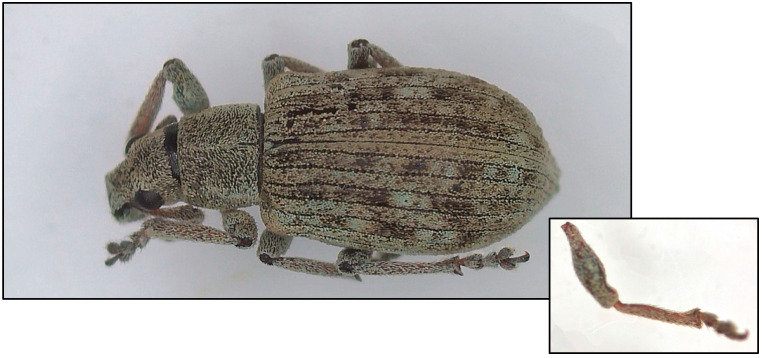
Dorsal view of adult *Polydrusus tibialis*, with an additional focus on the tibia.

*Polydrusus tibialis* is 3.2–4.9 mm in length, with a slender body and a slightly curved rostrum (Figure 1). The body, head, and legs of an adult *P. tibialis* are densely and thoroughly covered in scales. The elytra are striated with parallel lines (10 in total) that run the length of the body and may even have small punctures. The head is elongated, with large, oval compound eyes whereas the antennae are geniculate and clubbed. Finally, the legs are brown with color gradations of gray and bear distinct tibial spurs that characterize this species.

### 3.2. Biology

**Figure 2:**
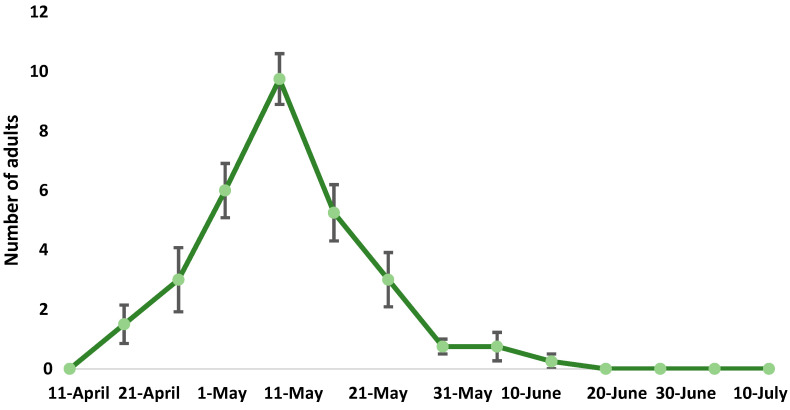
Mean number (± SE) of adult *Polydrusus tibialis* captured in traps in two peach orchards in the regional unit of Pella during the growing season of 2024.

**Figure 3:**
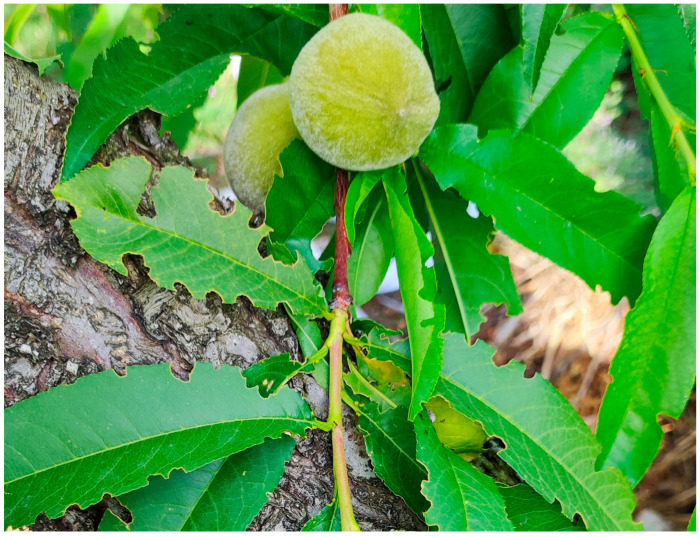
Characteristic serrated notches around the edge of the leaf blades due to the feeding of *Polydrusus tibialis* on the basal foliage of a peach tree.

**Figure 4:**
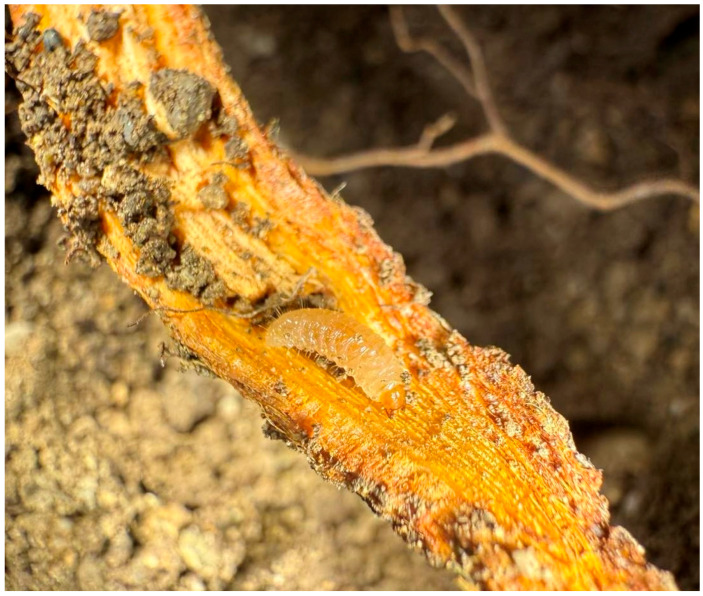
Larva of *Polydrusus tibialis* feeding on cambium/xylem tissues of a peach root.

*Polydrusus tibialis* has been reported to have one generation/year and overwinters as an adult or as a mature larva in the soil [17]. Our results confirm that it completes one generation/year in Northern Greece (Figure 2). According to our observations, adults emerge from overwintering sites in early April and until June, they climb the trunk of their host plants (in this case, peach), initially feeding on the basal foliage, causing characteristic serrated notches around the edge of the leaf blade (Figure 3). The damage by adult feeding has also been observed on newly formed fruit, immediately after petals fall. As affected fruits grow, they become deformed and unmarketable. After mating, adult females lay their eggs on the soil surface and hatched larvae feed on the rootlets and cambium/xylem tissues of roots (Figure 4).

The presence of *P. tibialis* in peach orchards in Northern Greece was first observed in the spring of 2024, in particular in the region of Central Macedonia. Also, serious infestation in commercial peach orchards was observed in the region of Thessaly (personal communication). This is in accordance with a previous study in Dalmatia (Croatia) that reported several curculionids, including two *Polydrusus* species, as members of the peach fauna [9]. Adult weevils were found feeding on peach leaves and shoots, causing noticeable damage to the foliage and the deformation of newly matured fruit. Subsequent inspections revealed that larvae were infesting the roots of young peach trees, leading to wilting and reduced growth.

Even though *P. tibialis* had been reported before in Greece [18], this is the first documented case of *P. tibialis* infesting peach trees in this region. Given the economic importance of peach cultivation in Northern Greece, the emergence of *P. tibialis* as a potential pest necessitates immediate attention from both researchers and growers. Further studies are required to assess the full impact of this pest on peach production and to develop effective management strategies.

## 4. Conclusions

The discovery of *P. tibialis* infesting peach orchards in Northern Greece marks a new challenge for local agriculture. This report highlights the need for the monitoring and development of integrated pest management strategies to mitigate the potential impact of this weevil on peach cultivation. Future research should focus on understanding the ecology of *P. tibialis* in peach orchards and exploring control measures that can effectively protect this economically important crop.

## Figures and Tables

**Table 1:** Locations of the peach orchards where pyramid traps were deployed for the sampling of *Polydrusus tibialis*.

Regional Unit	Location	Longitude	Latitude	Size (ha)
Pella	Aravissos	40.840690	22.251128	1.5
	Edessa	40.783034	22.045142	0.8

## Data Availability

The data that support the findings of this study are available from the corresponding author upon reasonable request.
